# Perceived barriers to reporting adverse drug events in hospitals: a qualitative study using theoretical domains framework approach

**DOI:** 10.1186/s13012-015-0302-5

**Published:** 2015-08-07

**Authors:** Fariba Mirbaha, Gloria Shalviri, Bahareh Yazdizadeh, Kheirollah Gholami, Reza Majdzadeh

**Affiliations:** Knowledge Utilization Research Center, Tehran University of Medical Sciences, No. 1547 North Karegar St, Enghelab Ave, Tehran, Iran; Iranian Pharmacovigilance Center, Food and Drug Organization, No. 24, Daneshkian Ave. Vali Asr St., Tehran, Iran; Research Center for Rational Use of Drugs, Faculty of Pharmacy, Tehran University of Medical Sciences, No. 92, Karimkhane Zand Street, 1584775311 Tehran, Iran; Knowledge Utilization Research Center; Epidemiology and Biostatistics Department, School of Public Health, Tehran University of Medical Sciences, Tehran, Iran

## Abstract

**Background:**

Adverse drug events (ADEs) are a major source of morbidity and mortality, estimated as the forth to sixth cause of annual deaths in the USA. Spontaneous reporting of suspected ADEs by health care professionals to a national pharmacovigilance system is recognized as a useful method to detect and reduce harm from medicines; however, underreporting is a major drawback. Understanding the barriers to ADE reporting and thereafter design of interventions to increase ADE reporting requires a systematic approach and use of theory. Since multiple theories in behavior change exist that may have conceptually overlapping constructs, a group of experts suggested an integrative framework called theoretical domains framework (TDF). This approach considers a set of 12 domains, came from 33 theories and 128 constructs, covering the main factors influencing practitioner behavior and barriers to behavior change. The aim of this study is to apply TDF approach to establish an evidence-based understanding of barriers associated with ADE reporting among nurses and pharmacists.

**Methods:**

A total of three focus group discussions were conducted; among them two consisted of nurses and one involved pharmacists. Discussions were guided by questions designed based on TDF. Transcriptions of discussions were then thematically analyzed, and detected barriers to reporting ADEs were categorized based on extracted themes.

**Results:**

A total of 34 nurses and pharmacists attended the group discussions. Six domains were identified to be relevant to barriers of ADE reporting in hospitals. These domains included “Knowledge,” “Skills,” “Beliefs about consequences,” “Motivation and goals (intention),” “Social influences (norms),” and “Environmental constraints.” We detected several barriers to ADE reporting, such as lack of knowledge of what should be reported, fear of punishment and criticism, lack of time, lack of teamwork, and lack of active support by hospital managements and other colleagues.

Based on detected barriers, “Cognitive and behavioral factors,” “Motivational factors and teamwork,” in addition to “Organizational processes and resources” could be targeted in designing appropriate interventions.

**Conclusions:**

Detection of barriers to reporting ADEs is necessary to design appropriate interventions. The TDF is a comprehensive approach that enables us to better understand barriers to behavior change in reporting ADEs.

## Background

Adverse drug events (ADEs) are major source of morbidity and mortality and represent a significant patient safety concern. According to World Health Organization (WHO) definition, ADE is “any untoward medical occurrence that may present during treatment with a pharmaceutical product but which does not necessarily have a causal relationship with this treatment” [1]. ADEs may involve adverse drug reactions (ADRs) and medication errors (MEs). ADR is “a response which is noxious and unintended, and which occurs at doses normally used in humans for the prophylaxis, diagnosis, or therapy of disease, or for the modification of physiological function” [1].

ME is “any preventable event that may cause or lead to inappropriate medication use or patient harm while the medication is in the control of the health care professional, patient, or consumer. Such events may be related to professional practice, health care products, procedures, and systems, including prescribing, order communication, product labeling, packaging, nomenclature, compounding, dispensing, distribution, administration, education, monitoring, and use” [2].

It has been estimated that ADEs are the forth to sixth leading cause of death in the USA [3]. Studies have shown that hospitalized patients are at considerable risk for experiencing ADEs, for example, a large meta-analysis of 39 prospective studies from US hospitals estimates that ADEs accounted for 4.6 % of all fatalities [4]. A study conducted in a Swedish population reported that 6.4 % of all fatalities in hospitals were related to ADRs [5].

Pharmacovigilance is “the science and activities relating to the detection, assessment, understanding and prevention of adverse effects or any other drug-related problem” [1]. Spontaneous reporting, as the backbone of pharmacovigilance, is “a system whereby case reports of ADEs are voluntarily submitted from health professionals and pharmaceutical manufacturers to the national regulatory authority” [1]. This method of reporting ADEs has been used for years by many national pharmacovigilance centers in the world, as a useful means for timely detection of ADEs in order to minimize drug-related morbidity and mortality [6].

### Pharmacovigilance activities in Iran

Iranian Pharmacovigilance Center (IPC) established spontaneous reporting system for collecting suspected ADEs in 1998 [7]. The ADE reports are submitted to IPC by health care professionals, from all over the country, through designed yellow cards. These reports include both ADRs and MEs. In order to improve hospital reporting of ADEs, IPC issued a guideline to all hospitals in the country. According to this guideline, each hospital designated a person (physician, nurse, or pharmacist) as drug safety officer (DSO). These DSOs from more than 600 hospitals were then trained by IPC. Based on the guideline, hospital staffs are free to report ADEs through DSOs or directly to IPC.

As a full member of WHO International Drug Monitoring Program, IPC has now collected more than 35,000 reports of ADEs from all around the country. However, like many other national centers in the world, it faces underreporting as a major drawback of spontaneous reporting [8]. It is estimated that only 2–4 % of all ADEs and 10 % of serious ones are reported [9]. Underreporting threatens drug safety due to delay in detecting drug-related problems, making it difficult to evaluate benefit-risk balance of medicines. Studies have explained many reasons for underreporting, including fear of litigation, lack of knowledge (on what and how to report), lack of confidence about drug-reaction relationship, lack of interest, lack of time, wrong beliefs (e.g., all approved medicines for marketing are safe), and unavailability of yellow card [10]. Other studies added attitude and motivational factors to the above list [11]. However, there are limited studies that have evaluated barriers for reporting ADEs based on theories of behavior change [12].

### Understanding the barriers to ADE reporting based on theoretical domains framework

Although health care can be improved at different levels of the health system, one important intervention is to support individual health professionals to modify their behavior based on evidence [13]. The behavior evaluated to be modified in this study is reporting ADEs. A thorough understanding of the barriers of this behavior is an important first step in the development of strategies to address the barriers which may lead to effective interventions and increasing reporting and consequently reducing ADEs and improving patient safety.

Understanding the barriers to ADE reporting and thereafter design of interventions requires a systematic approach and use of theory [14, 15]. With a better theoretical understanding of healthcare professional behaviors, the likelihood of success of interventions to change behavior may be elevated. Theory can be used to understand the factors and barriers that might influence the targeted behavior change, to identify possible techniques that could be utilized to change clinical behavior [16], and to clarify how such techniques might work [17]. But multiple theories and frameworks of individual and organizational behavior change exist, and often these theories have conceptually overlapping constructs [13, 18]. In addition, only a few of these theories have been tested in robust research in healthcare settings. There needs to be a systematic approach for determining the relevant theories (from all available theories) which predict behavior or behavior change most comprehensively [19, 20].

Thus, theory selection needs to be based on a comprehensive theoretical assessment of the targeted behavior, otherwise we run the risk of (1) missing relevant theoretical constructs or including the irrelevant ones and (2) designing interventions based on theories with overlapping theoretical constructs [17, 21, 22]. This would make identifying the specific processes involved in behavior change very difficult.

As a solution to these problems, a group of experts, including psychological theorists, health psychologists, and health service researchers, came up with an integrative framework called theoretical domains framework (TDF) [16]. These experts identified psychological and organizational theories relevant to health professional behavior change. Then they came up with a set of 12 domains covering the main factors influencing practitioner behavior and barriers to behavior change which included: Knowledge; Skills; Social/professional role and identity; Beliefs about capabilities; Beliefs about consequences; Motivation and goals; Memory, attention, and decision processes; Environmental context and resources; Social influences (norms); Emotion; Behavioral regulation; and Nature of the behaviors. Since these theoretical domains came from 33 theories and 128 constructs that explain health-related behavior change, they may result in thorough understanding of the targeted behavior, its barriers, and consequently comprehensive intervention programs.

TDF has extensively been used to identify barriers to change in healthcare in order to develop interventions [21, 23–30]. Examples of these studies include investigation of the barriers and levers to hand hygiene [24] and assessment of theoretical domains relevant to blood transfusion practice in different contexts [25]. Also in Australia, TDF has been used to identify the barriers to the implementation of evidence-based guidelines for acute low back pain [23, 31] and develop theory-informed behavior change interventions [17]. Furthermore, in Denmark, it has been used to enhance implementation of tobacco use prevention and counseling guidelines among dental providers [27].

The aim of the current study is to apply a broad comprehensive theory-based approach (utilizing TDF) to establish an evidence-based understanding of the processes and barriers associated with ADE reporting among nurses and pharmacists. Therefore, theoretical domains that should be considered by an effective intervention program to improve ADE reporting were identified. The result of this study, when used to design tailored interventions in improving ADE reporting, could also add to the science of implementation research.

## Methods

### Study participants and design

This is a qualitative study based on focus group discussions (FGDs). The IPC list of DSOs in Tehran hospitals was used as the sampling frame for sample selection. There were 117 DSOs from 117 hospitals in Tehran, among them 60 were nurses, 51 pharmacists, and 6 physicians. Nurses reported most ADEs of the total registered cases in IPC database, followed by pharmacists and physicians. During a 2-year period, IPC had received 3674 ADE reports sent by nurses, followed by 750 reports by pharmacists, and 327 reports by physicians, so we selected nurses and pharmacists as the groups of choice to evaluate their opinion about barriers to ADE reporting. We excluded hospitals in which physicians were DSO since there were limited ADE reports received from those hospitals. Then we first classified the remainder 111 hospitals by the job of DSOs (i.e., nurses or pharmacists), then by type of hospital (private or public), ADE reporting rate (good or weak reporting hospitals based on IPC database), size of the hospital (small or large hospitals based on number of beds), and hospitals from both affluent and disadvantaged areas of the city. We selected 45 DSOs that at least one person from each hospital group was selected to be included in each FGD. The selected DSOs were invited to participate in FGDs. A total of 3 FGDs were conducted in summer of 2013, among them 2 consisted of nurses (30 persons) and 1 involved just pharmacists (15 persons). In order to get broad information about barriers to ADE reporting, we invited both DSO and non-DSO nurses to participate in the two FGDs. But pharmacists were all DSOs since IPC had not received considerable number of reports from those who were not DSO. Informed consent was obtained from all FGD participants.

In order to ensure consistency, the same facilitators conducted the FGDs. A pharmacist expert in pharmacovigilance along with a medical sociologist and an epidemiologist expert in knowledge translation and exchange (KTE) conducted all FGDs. Each session was about 90 min. The discussions were recorded, and one of the facilitators took notes in the sessions.

### Discussion questions

A set of questions was designed based on 12 different domains of TDF developed by Michie et al. [16]. The discussion questions were first piloted in two different interviews and revised based on the results. The final version of the questions was discussed in focus groups. During and at the end of discussions, FGD participants were asked to add more comments on discussed questions or domains. They were free to add these additional points during FGD or to contact the research group later via phone or email.

### Data analysis

A verbatim transcript of each FGD was prepared from the tape recording of the sessions. Transcription of all audio tapes was done externally (by a paid company), and one of the authors (GS) reviewed them for accuracy and completeness. To maintain anonymity, names of people and hospitals were removed from transcripts. Two of the researchers read all three FGD transcripts multiple times, on their own. They coded the transcripts based on the 12 domains of TDF and performed thematic analysis. Both inductive and deductive approaches were used to make sure no theme was missed. Specific themes were extracted and then categorized according to 12 domains of TDF, with their related subthemes. To assess agreement between two researchers, all extracted themes and subthemes were reviewed in a meeting. The two researchers had high agreement (>90 %). Disagreements were discussed and a final theme or subtheme was chosen.

### Ethics

The study has been approved by the Institutional Review Board of Tehran University of Medical Science which follows the Helsinki Declaration.

## Results

Among 45 invited persons, 34 nurses and pharmacists attended the FGDs. Statistics on how many nurses and pharmacists attended FGDs can be found in Table 1. Based on FGDs, we excluded domains that involved irrelevant themes. Also, whenever we identified overlapping in detected themes in different domains, we excluded irrelevant domains. We discovered the same themes using inductive and deductive approaches, so we did not report them separately. A total of six domains were identified to be relevant to barriers and facilitators of ADE reporting in hospitals. These domains included Knowledge, Skills, Beliefs about consequences (anticipated outcomes/attitude), Motivation and goals (intention), Environmental context and resources (environmental constraints), and Social influences (norms). We have discussed the details of the method for selection of domains elsewhere (paper in progress). Sixteen different themes were extracted from transcripts of FGDs. The identified themes across relevant domains, and related questions, are demonstrated in Table 2.

**Table 1:** Statistics on how many nurses and pharmacists attended FGDs

Focus group	Participants occupation	Number of invited persons	Number of attendees
First FGD	DSO nurses	15	12
Second FGD	DSO pharmacists	15	10
Third FGD	Non-DSO nurses	15	12

**Table 2:** Questions related to different domains and identified themes

Domain	Questions	Themes
Knowledge	What do you know about ADE reporting system in the country?	Awareness of ADE reporting system
	How do you define ADR?	Awareness of ADE reporting guideline
	How do you define ME?	Awareness of what should be reported
		Awareness of ADE definitions
Skills	What is the difference between ADR and ME?	Distinguish between ADR and ME
	Is reporting ADEs difficult for you?	Reporting difficulty
Beliefs about consequences	What are the outcomes of reporting ADEs? ( Both positive and negative outcomes)	Positive outcomes
		Negative outcomes
Motivation and goals	How motivated are you to report ADEs?	Motivation to report
	What are the incentives in reporting ADEs?	Incentives
	Do you regularly have other activities or goals that might interfere with your reporting ADEs?	Interference with other goals and activities
Environmental context and resources	To what extent do physical factors or resources facilitate or hinder your reporting ADEs?	The impact of physical factors or resources on reporting ADEs
	How does time constraint impact your reporting ADEs?	Time constraint
Social influences (norms)	Are ADEs actively reported by other health professionals in your hospital?	ADE reporting by other hospital staff
	Do hospital managers and your colleagues approve your reporting ADEs?	Reporting approval by hospital managers and colleagues
	Is there anyone who disapproves or opposes your reporting ADEs in your hospital?	Disapproval of ADE reporting

### Knowledge

Most nurses and pharmacists were aware of ADE reporting process in their hospitals; however, they were not well informed of reporting guidelines on ADE reporting. They could not provide a comprehensive definition of ADE, ADR, or ME in accordance with the national guideline for reporting ADEs. Here are some of the definitions they offered:

“ADRs are related to the medication but MEs are errors in administration of the medication”

“MEs are not part of ADEs.”

Nurses and pharmacists in all three FGDs stated that serious ADEs, in addition to cutaneous ADEs, were more frequently reported in their hospitals compared to other types of ADEs. They also mentioned that MEs which resulted in patient harm were mostly reported. Most focus group participants believed that ADEs which were common, certain, known, or preventable should be reported. However, there was some controversy in reporting common and routine ADEs. Here are some of their comments:

“We only report common ADEs and those that are mentioned in the brochures.”

“If we report all ADEs, physicians will seriously be limited [in prescribing medicine].”

“We do not report routine ADEs otherwise we have to spend most of our time filling up forms [to report ADEs].”

### Skills

Nurses and pharmacists who participated in the focus groups did not feel reporting ADEs was difficult to do. However, nurses found reporting ADRs less controversial than reporting MEs.

Participants, in general, were not able to differentiate between ADR and ME in practice:

“MEs are errors in drug use which do not lead to patient harm.”

“MEs happen before drug consumption while ADRs occur after using medication.”

### Beliefs about consequences (anticipated outcomes/attitude)

In general, focus group participants expected many positive consequences by reporting ADEs. The most reported positive outcomes of ADE reporting included the following: reducing drug-related morbidity and mortality, increasing scientific knowledge of health professionals, improving quality of medicines, decreasing therapeutic cost, shortening hospital stay, replacing problematic drugs with safer alternatives, detecting counterfeit medicines, improving pharmacotherapy, preventing ADEs in other patients, reducing overuse of medicines, identifying precautions for drug use, preventing drug interactions, and improving patient safety. Some of the participants’ comments are listed below:

“A good example is the problems we encountered following injection of ceftriaxone. When we became familiar with its adverse reactions, we realized that we should be more careful.”

“In my opinion, the most important consequence is that we can identify problems in manufacturing medicinal products.”

On the other hand, a few participants pointed out the following negative consequences: punishment of individuals involved in occurring ADE or recall of a beneficial medicine from the market by mistake. Here are some examples of the participants’ comments:

“If what is reported is not a real ADE, the suspected medicine may be recalled by mistake.”

“There may not be negative consequences for us but the manufacturers may incur some unjustified expenses.”

### Motivation and goals (intention)

Most nurses and pharmacists in the focus groups reported that they were motivated to report ADEs. That was an expected result since most participants were DSOs. However, there were few individuals who mentioned that they might not choose to report because of lack of feedback from the pharmacovigilance center. They thought it was just a waste of time since they did not see a positive effect from their reporting. Here is an example comment:

“The problem is that we report ADEs but do not get any feedback.”

Some FGD participants referred to lack of motivation for reporting ADEs in nursing staff except for DSOs, especially the young generation. In addition, fear of criticism and punishment was suggested as a barrier to reporting ADEs by nursing staff. Some of the comments in this domain were as follows:

“If nurses think that we [the supervisors] will criticize them, they won’t report.”

“The young generation is more relaxed and a bit lazy. They are not well educated in ethical concerns and they are not patient enough to fill in yellow cards.”

“They [young nurses] don’t seem to be motivated.”

“A young nurse fresh out of college to tell me [the supervisor] that I should do the job for the patient myself, in my opinion, they need ethical training. They don’t give the patient their medication and when questioned they say that they were out of that medicine!”

“If nurses were not under pressure of their supervisors to report, they would not report ADRs.”

Some participants believed that new activities related to hospital accreditation took a lot of their time and made them not want to take on any other responsibility such as ADE reporting. One participant commented the following:

“Another reason is that hospital staff has been urged to do lots of new activities in a short period of time and everyday something is added to the list so nurses are all involved in this issue and show resistance to take on any additional activity. When they are asked to do a minute thing, they react by saying that they are exhausted. I think hospital accreditation staff has a lot to do with it.”

Many FGD attendees outlined incentives as a major factor in reporting ADEs. Only a handful of hospitals currently seemed to have incentives for ADE reporting such as gift certificates. Interestingly, the majority of hospitals offered incentives for internal reporting of MEs. Here are some of the comments in this regard:

“We should always encourage ADR reporting, even a nurse should understand that potential bonuses is due to her ADR reporting”

“The [pharmacovigilance] center should send a letter to say thank you for your efforts.”

“Those with lots of ADE reporting [in her hospital] receive incentives from the head nurse.”

“I have seen that nurses committed some MEs, they reported their own errors and have been praised for doing that and received incentives.”

### Environmental context and resources (environmental constraints)

A major concern outlined by nurses and pharmacists was lack of time for reporting ADEs. A number of nurses pointed out that completing yellow cards for ADE reporting took a long time. Other mentioned barriers were complicated and time-consuming administrative procedures in reporting process and limited access to appropriate equipment and resources for submitting ADE reports. Lack of adequate human resources and fast turnover rate of nurses were other mentioned hampering factors. They mentioned that fast turnover rate of nurses led to missed trained nurses, and they had to repeat educational programs on ADE reporting from the beginning. Here are some examples of the comments:

“When two nurses are in charge of 30 patients, they really don’t have time to do such things [report ADEs]. I, myself, observe ten cases of ADE but don’t have time to report even one case. I report them all once and say for example that ten patients have developed same ADE following consumption of a particular medicine, because I have no time to fill in a separate yellow card for each patient.”

“Sometimes it takes half an hour just to fax a yellow card to ADR Center.”

Participants in all three FGDs suggested that involvement of clinical pharmacists who, in their opinion, were experts in recognizing ADEs could really benefit ADE reporting in hospitals. They also stated that easy access to yellow cards could facilitate ADE reporting.

### Social influences (norms)

FGD participants believed that good social relations among supervisors and nursing staff increased ADE reporting. Here are some of the comments:

“If we [supervisors] present and explain the requirements [for ADE reporting] appropriately, they [the staff] will collaborate with us.”

“Not everybody wants to get involved; it really depends on type of interaction between staff and supervisors.”

One of the major barriers for ADE reporting, to them, was lack of collaboration between nurses, pharmacists and physicians. They named teamwork an essential component of ADE reporting.

All three focus groups reported that hospital executive administrators approved ADE reporting; however, they did not do anything to facilitate it. There were no incentives or punishment systems in place to improve ADE reporting. Supervisors and other health care professionals in their hospitals also approved ADE reporting while they might not play an active role. Here is one of the comments:

“Hospital top management surely approves ADE reporting; however they do not get involved. There are no incentives or punishments in regard to reporting in place.”

Generally, nurses and pharmacists in the focus groups stated that there were no individuals or groups of people who really opposed ADE reporting in hospitals. However, some nurses were not motivated enough to report ADEs. They accounted fear of punishment and limited time for completing ADE-reporting procedure as reasons for lack of motivation in ADE reporting. These unmotivated nurses did not agree with ADE reporting in hospitals. Below some of these comments are listed:

“Nobody openly announces that they are against reporting, although there are some nurses who are not enough motivated.”

“A few times, even the completed yellow card was not sent [by nurses because of lack of motivation], I [DSO] had to follow it up.”

## Discussion

We identified several barriers to reporting ADEs in this study. Identified barriers in each detected domain are shown in Table 3. Also, examples of suggested interventions to remove these barriers are listed in the same table [32].

**Table 3:** Identified barriers and examples of appropriate interventions in different domains

Domain	Identified barriers	Example of appropriate interventions [32]
Knowledge	Lack of knowledge of what should be reported	Information delivery methods adopted to individual needs
	Lack of knowledge of definitions	
	Lack of knowledge of guideline	
Skills	Lack of skills in differentiating ADRs and MEs	Provide education to improve competency
Beliefs about consequences	Fear of punishment and criticism	Provide education on consequences
Motivation and goals	Lack of feedback	Provide more feedback such as timely alerts
	Lack of motivation	Provide information about impact of reporting, social influence (e.g., provide a role model)
	Heavy workload	Training on time management, provide help
	Lack of incentives	Provide appropriate incentives
Environmental constraints	Lack of sufficient human resources	Establish specific department for drug safety
	Lack of sufficient time for reporting	Revise reporting procedures
	Complicated yellow card	Revise and redesign yellow card
	Complicated administrative reporting procedure	Redesign reporting procedure
	Lack of reporting facilities	Provide appropriate facilities
	Lack of clinical pharmacist	Recruit and train clinical pharmacist
	No access to yellow cards	Easy access to yellow cards
Social influences	Lack of teamwork	Training to change group processes
	Lack of active support by hospital management and other colleagues	Organize social influence (provide support )

In the category of “Knowledge” domain, the results of this study revealed that there was low awareness of what should be reported among nurses and pharmacists in the studied hospitals. According to many of the study participants, ADEs should be reported when they are common, certain, known, or preventable. This finding is in accordance with other studies [33–35]. Participants’ lack of awareness about reporting requirements was partly due to their unfamiliarity with the guidelines. Interventions should focus on special education and training, including the published guidelines, since they can find necessary information on what and how ADEs should be reported. We found that all participants were aware of the pharmacovigilance system in the country. This is in contrast with studies that discovered that not all hospital physicians and nurses had enough knowledge of the existence of ADE reporting system in their country [33, 35]. Cognitive and behavioral factors should be targeted in designing appropriate interventions for this level of barriers such as those suggested in Table 3.

With regard to “Skills” domain, according to study participants, nurses and pharmacists did know how to report ADRs and found it fairly easy to do. However, reporting MEs was not as easy for nurses. This formed another barrier for reporting as lack of skills in differentiating ADRs and MEs so that nurses moved towards non-reporting of MEs to IPC. We discovered that nurses were somewhat uneasy about reporting MEs if they felt that confidentiality with reporter identity was not respected. Confidentiality with patients’ data has been identified as a matter of importance in another study conducted in a Spanish tertiary teaching hospital [10]. Providing education such as workshops to improve nurses’ skills in reporting MEs is suggested as an appropriate intervention.

In the “Beliefs about consequences” domain, problems of legal liability and possible judicial claims were detected as a potential obstacle to reporting ADRs in Spanish study [10]. However, we found fear of punishment and litigation of reporter or hospital staff involved in occurring ADE as a major barrier for reporting ADEs. The respondents perceived this barrier as a probable negative outcome of ADE reporting. Another problem was that they were trained to report MEs internally and excluded them from reporting to IPC. We found that there is a need to educate nurses and pharmacists that there is no punishment for reporters, and reporter identity is confidential.

As a motivational factor, we concluded that incentives may have a considerable impact on increasing ADE reporting in hospitals. This factor has been discussed in other studies [36–38]. These studies have shown that financial incentives could have positive consequences and may lead to increased reports of ADEs. However, as suggested by our study participants, nonfinancial incentives could also be offered. Consequently, interventions to promote ADE reporting should include well-defined incentives for those who report ADEs, provided by different levels of health system management (e.g., hospital management, IPC, etc.). For example, drug safety annual award for high-quality ADE reporting can be considered.

By detecting lack of teamwork as a “Social influences (norm)”-related barrier, the results of our study indicated that interventions that target teamwork are also necessary to improve ADE reporting. Nurses reported very low level of collaboration with physicians and pharmacists in reporting ADEs. They mentioned that they did not have enough collaboration of physicians in diagnosis or confirmation of ADEs. Similarly, they complained of lack of collaboration of pharmacists in sharing information on side effects of drugs or their medicinal consultation. A possible solution to this obstacle is to improve participation of pharmacists in detecting and reporting ADEs. We conclude that reporting ADEs in hospitals needs a strong team and teamwork in which responsibilities of each member of the group is well defined. This finding was new to us since we did not find similar findings in other studies.

Lack of active support by hospital managements and other colleagues was another barrier detected in “Social influences (norms)” domain. The results of this study showed that reporting ADEs was socially influenced by culture of reporting in the hospital. Nurses did not have role models among supervisors and higher management and did not feel they were socially supported in their hospitals for reporting ADEs. Hospital executives did not have promotion of ADE reporting on their agenda and did not seem to have different plans for the future. The culture of reporting was not well established in the hospitals, making it difficult for nurses to overcome perceived drawbacks of reporting, such as confrontation with supervisors and other colleagues. To overcome this obstacle, interventions that target hospital managers are helpful as they are at the top of the pyramid of decision-making.

Barriers related to “Environmental constraints” including lack of human resources as well as technical facilities were identified as barriers to reporting ADEs in our study. As an example for human resources, utilization of additional clinical pharmacists as reporting facilitators was suggested by the studied participants. Studies have shown that the presence of a clinical pharmacist in a hospital results in increased ADE reporting [39, 40]. Lack of reporting facilities and complicated administrative procedures were noted as problematic to the ADE reporting in this study. Participants declared that lack of facilities such as easy access to fax, the availability and easy access to yellow cards, and an internet-based reporting system could have negative impact on ADE reporting. Consistent with other studies [10, 41], lack of sufficient time was identified in this study as a major barrier for ADE reporting. A comprehensive intervention should include time management training and strategies such as revising and shortening of reporting procedures. For instance, yellow cards, as well as detailed administrative procedure of reporting in hospitals, have to be reviewed for necessary changes.

## Limitations of the study

We faced some limitations in this study. The first one was low rate of ADE reporting by physicians and non-DSO pharmacists, and that was why they were not included in FGDs.

Another limitation, as in any FGD, was that some participants were more willing to speak and present their ideas than others. As a result, some participants might have refrained from presenting their opinions if others had already stated similar ideas. In order to overcome this limitation, at the end of each discussion topic, we asked the participants, whether they agreed or disagreed with the discussions.

There are numerous terms in the field of pharmacovigilance, each one involved special aspect of what should be reported. This variety of terminology led to another limitation in choosing appropriate terms for evaluating participants’ knowledge about what should be reported. As we mentioned above, ADR and ME are two elements of ADEs. It was important to us to assess the ability of participants to distinguish between these two words, so we asked them to define ADR and ME, separately. The answer to this question could reveal how study participants think about the concept of what should be reported or the concept of ADE. So we preferred not to confuse them by asking the definition of several words, instead we asked about the components of ADE which were ADR and ME.

## Conclusions

Detection of barriers to reporting ADEs by nurses and pharmacists is necessary to design appropriate interventions. The main barriers detected in this study were related to six domains of Knowledge, Skills, Beliefs about consequences, Motivation and goals, Environmental constraints, and Social influences (norms). Some of the detected barriers in this study were previously determined by other studies conducted in different settings, such as lack of knowledge or skills in different aspects of ADE reporting, fear of punishment and criticism, and/or lack of time. However in this study, TDF helped us explore some barriers such as problems in team working which was not identified in similar studies. We believe that TDF is a comprehensive approach that enables us to better understand and classify barriers to behavior change in reporting ADEs. Classification of barriers based on different psychological domains could be effective in mapping suitable interventions to detected barriers. Appropriate interventions should be tailored and implemented based upon identified barriers in each of the related domains.

## Abbreviations

ADE: adverse drug event
ADR: adverse drug reaction
DSO: Drug Safety Officer
FGD: focus group discussion
IPC: Iranian Pharmacovigilance Center
KTE: Knowledge Translation and Exchange
ME: medication error
TDF: theoretical domains framework
WHO: World Health Organization
